# Thymic Atypical Carcinoid Mimicking Recurrent Type A Thymoma on Frozen Section: A Diagnostic Pitfall Resolved by Intraoperative Imprint Cytology

**DOI:** 10.7759/cureus.103253

**Published:** 2026-02-09

**Authors:** Thao T Nguyen, Kazuki Fujita, Motona Kumagai, Daisuke Hoshi, Sohsuke Yamada

**Affiliations:** 1 Department of Pathology and Laboratory Medicine, Kanazawa Medical University, Ishikawa, JPN; 2 Department of Pathology, Kanazawa Medical University Hospital, Ishikawa, JPN; 3 Department of Pathology II, Kanazawa Medical University, Ishikawa, JPN; 4 Department of Pathology, Kanazawa Medical University, Ishikawa, JPN; 5 Department of Oncologic Pathology, Kanazawa Medical University, Ishikawa, JPN

**Keywords:** anterior mediastinum, atypical thymic carcinoid, imprint cytology, thymic epithelial tumors, thymic neuroendocrine neoplasms, type a thymoma

## Abstract

Thymic neuroendocrine neoplasms (tNENs) are rare anterior mediastinal tumors with aggressive behavior and can be misdiagnosed as type A thymoma on small biopsies or intraoperative frozen sections, although accurate distinction is critical for prognosis and management. Type A thymoma, while generally considered a low-grade malignant tumor with a favorable prognosis, comprises a small subset that exhibits aggressive features and develops distant metastases after surgical resection; these tumors are classified as atypical type A thymomas. A 72-year-old woman had a history of resected atypical type A thymoma two years earlier. Surveillance computed tomography revealed a 15-mm mediastinal nodule located anterior to the superior vena cava with intense fluorodeoxyglucose uptake on positron emission tomography-computed tomography. Frozen sections showed a proliferation of small- to medium-sized polygonal and short spindle cells arranged in solid nests and trabeculae without a lymphocyte-rich background, and were interpreted as recurrent atypical type A thymoma. In contrast, imprint cytology demonstrated monomorphic small- to medium-sized tumor cells with round to oval nuclei, finely granular “salt-and-pepper” chromatin, inconspicuous nucleoli, loose cohesion, and scattered rosette-like structures, strongly suggesting a tNEN. Permanent sections revealed nests, trabeculae, and rosettes of small- to medium-sized polygonal cells with granular chromatin and approximately four mitoses per 10 high-power fields, without large confluent necrosis. Immunohistochemistry showed diffuse positivity for CD56, chromogranin A, synaptophysin, and insulinoma-associated protein 1; a Ki-67 index of about 20%; negativity for CD5, CD117 (c-KIT), p63 (TP63), CK5/6, and CD20; and the absence of TdT/CD99-positive immature T cells, supporting a diagnosis of thymic atypical carcinoid. This case highlights the complementary value of imprint cytology and an appropriate immunohistochemical panel, in addition to frozen sections, in avoiding misclassification of tNENs as type A thymoma.

## Introduction

Thymic epithelial tumors (TETs) are extremely rare, with an annual incidence of only 0.23-0.30 per 100,000 population, but they represent the most common primary tumors of the anterior mediastinum [1]. TETs comprise three major categories: thymomas, thymic carcinomas, and thymic neuroendocrine neoplasms (tNENs) [2-4].

tNENs account for only 2-5% of all TETs [4,5]. According to the 2021 WHO classification, tNENs are categorized as typical carcinoid (TC), atypical carcinoid (AC), large-cell neuroendocrine carcinoma, and small-cell carcinoma [2,5,6]. Among these, AC is the most frequent subtype, accounting for approximately 40-50% of reported cases [6].

Thymoma is another major subgroup of TETs [2-4]. Type A thymoma, while generally considered a low-grade malignant tumor with a favorable prognosis, comprises a small subset (approximately 3.8%) that exhibits aggressive features and develops distant metastases after surgical resection; these tumors are classified as atypical type A thymomas [7].

Accurate discrimination between AC and atypical type A thymoma is clinically important because tNENs generally behave more aggressively and require different therapeutic strategies and follow-up [8]. Intraoperative imprint cytology is a simple technique that allows clearer assessment of nuclear details and fine microarchitectural features than frozen sections [9].

In this report, we describe a rare case of thymic AC in a patient with a prior history of atypical type A thymoma, in whom the intraoperative frozen section favored recurrent thymoma, whereas the imprint cytology strongly suggested a tNEN, thereby highlighting the diagnostic value of this technique.

## Case presentation

**Figure 1:**
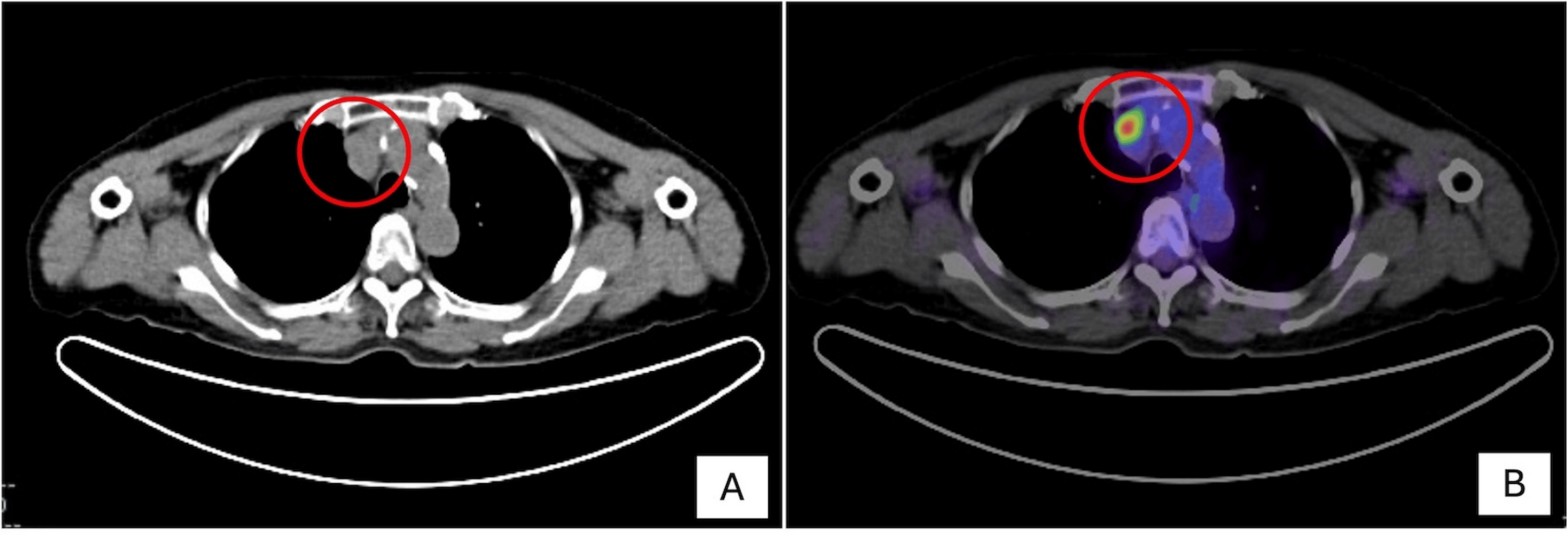
Imaging findings. Axial computed tomography (A) and positron emission tomography-computed tomography (B), displayed according to standard radiological convention, showing intense fluorodeoxyglucose uptake in the anterior mediastinal lesion.

A 72-year-old woman had a history of resected atypical type A thymoma two years earlier. Surveillance computed tomography (CT) detected a nodule in the anterior mediastinum, located anterior to the superior vena cava (Figure 1A). Positron emission tomography-computed tomography (PET-CT) demonstrated intense fluorodeoxyglucose (FDG) uptake in this lesion (Figure 1B). At the time of admission, routine laboratory investigations, including complete blood count, inflammatory markers, serum biochemistry, and tumor markers, were within normal limits; however, the anti-acetylcholine receptor antibody level was elevated (7.3 nmol/L) in the absence of clinical features of myasthenia gravis. Surgical resection was performed via a minimally invasive approach using conventional video-assisted thoracoscopic surgery, with intraoperative frozen-section consultation.

**Figure 2:**
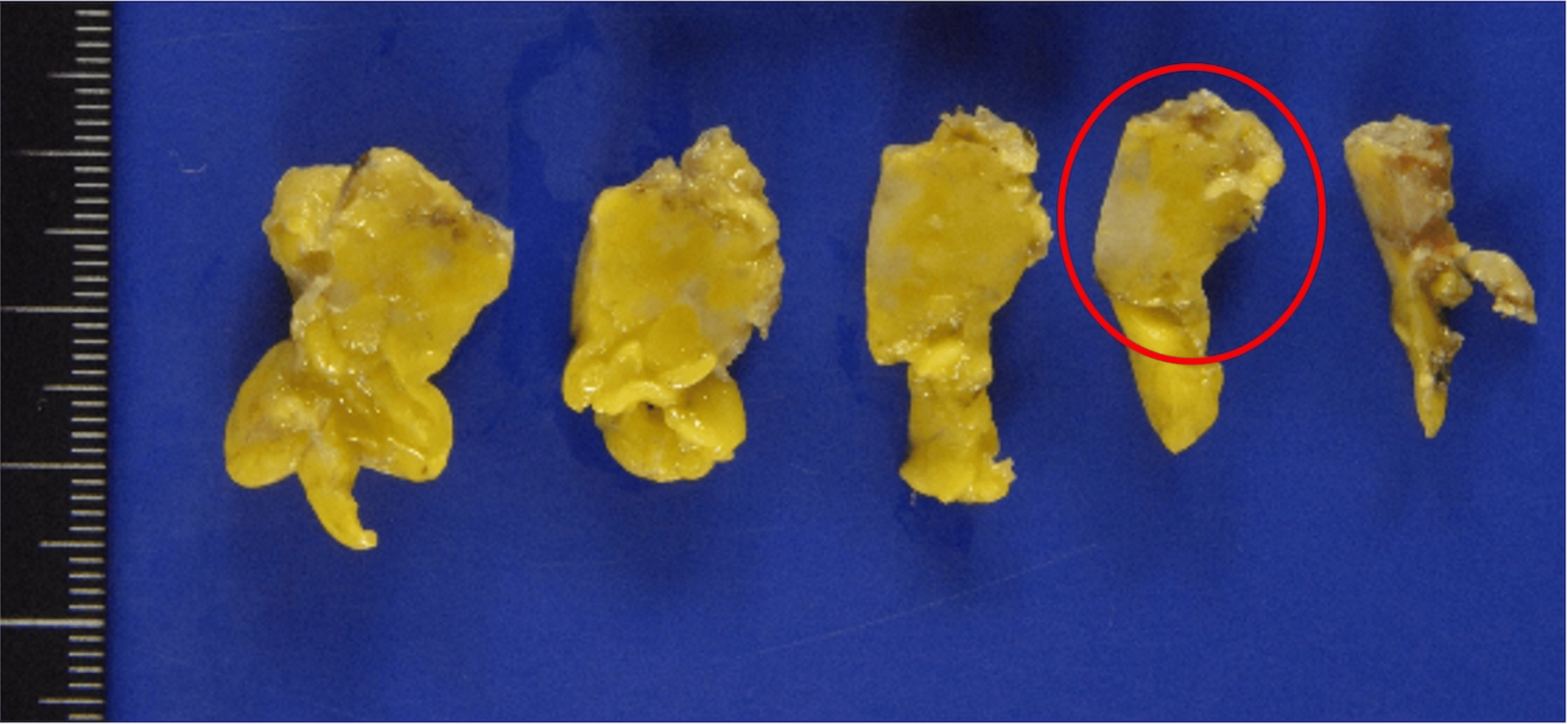
Gross examination. The surgical specimen was cut into multiple pieces during resection. The lesion appeared as a firm, yellow-whitish nodule in the anterior mediastinum. One representative piece is highlighted (circled).

**Figure 3:**
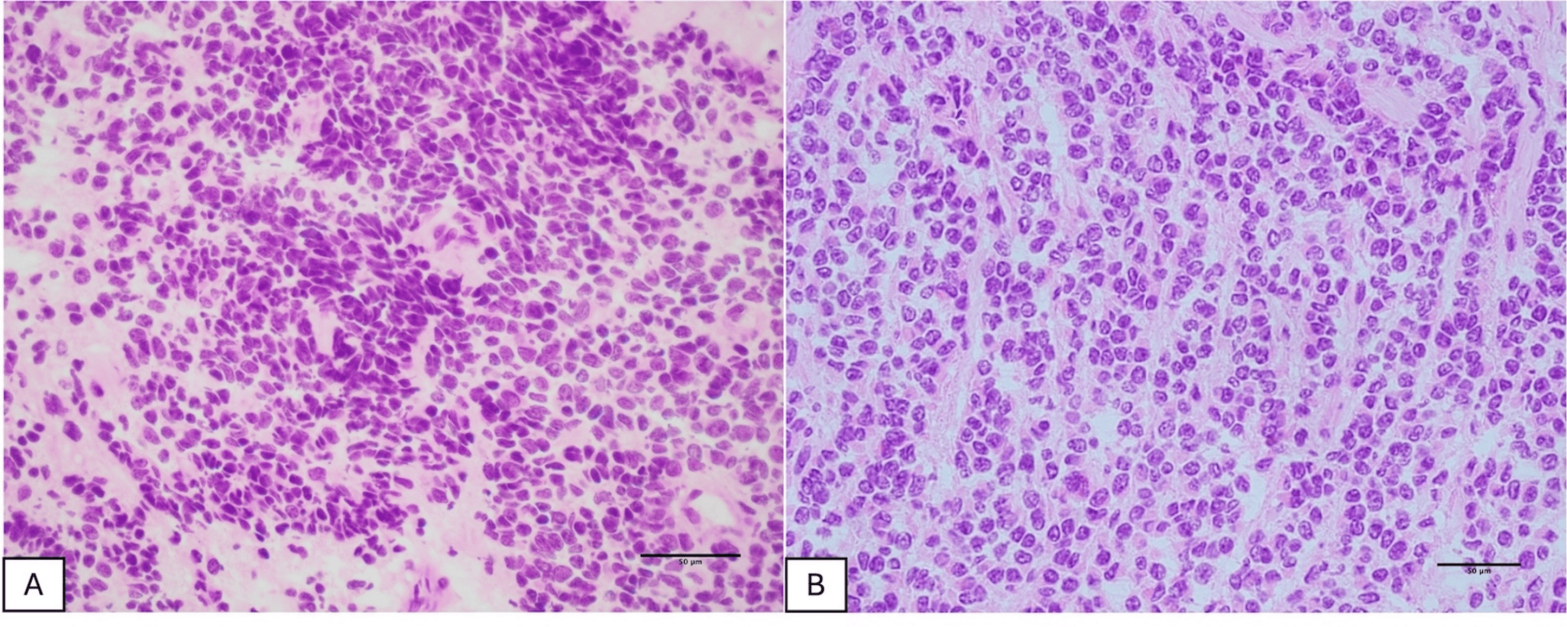
Frozen-section hematoxylin and eosin findings (×40). (A) A densely cellular proliferation of short spindle tumor cells arranged in solid sheets. (B) Polygonal tumor cells with round to nearly round nuclei arranged in trabecular cords, with fine chromatin and no admixed lymphocytes.

On gross examination, the lesion was a firm, yellow-whitish nodule measuring approximately 15 mm, with ill-defined borders within the surrounding adipose tissue (Figure 2). On the frozen hematoxylin and eosin sections used for intraoperative consultation, there was a densely cellular proliferation of short spindle cells arranged in solid sheets, with hyperchromatic nuclei, intermixed were tumor cells with round to nearly round nuclei (Figure 3A). In another area, polygonal tumor cells with round to nearly round nuclei were observed, proliferating in trabecular cords. The nuclear chromatin was fine, with only mild nuclear atypia, and no admixed lymphocytes were identified (Figure 3B). In the context of the patient’s medical history, the new lesion was interpreted on intraoperative frozen section as recurrent type A thymoma.

**Figure 4:**
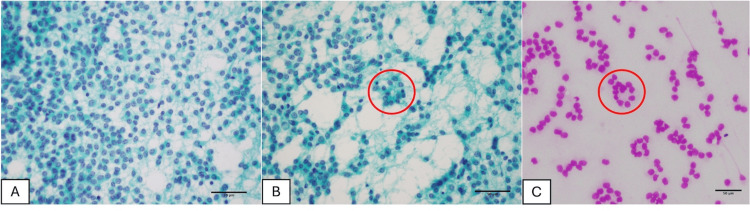
Imprint cytology of the tumor. (A) Polygonal tumor cells showing finely granular “salt-and-pepper” chromatin (Papanicolaou stain, ×40). (B, C) Scattered rosette-like structures (Papanicolaou and Giemsa stains, ×40).

Imprint cytology was simultaneously performed. Papanicolaou-stained smears revealed small- to medium-sized, monomorphic polygonal tumor cells with delicate cytoplasm and round to nearly round nuclei showing finely granular “salt-and-pepper” chromatin. Nucleoli were small or inconspicuous, mitotic figures were not identified, and nuclear atypia was minimal (Figure 4A). Notably, scattered rosette-like structures were observed on both Papanicolaou- and Giemsa-stained smears (Figures 4B, 4C). Based on these cytologic features, the lesion was considered more consistent with a tNEN than with a thymoma.

**Figure 5:**
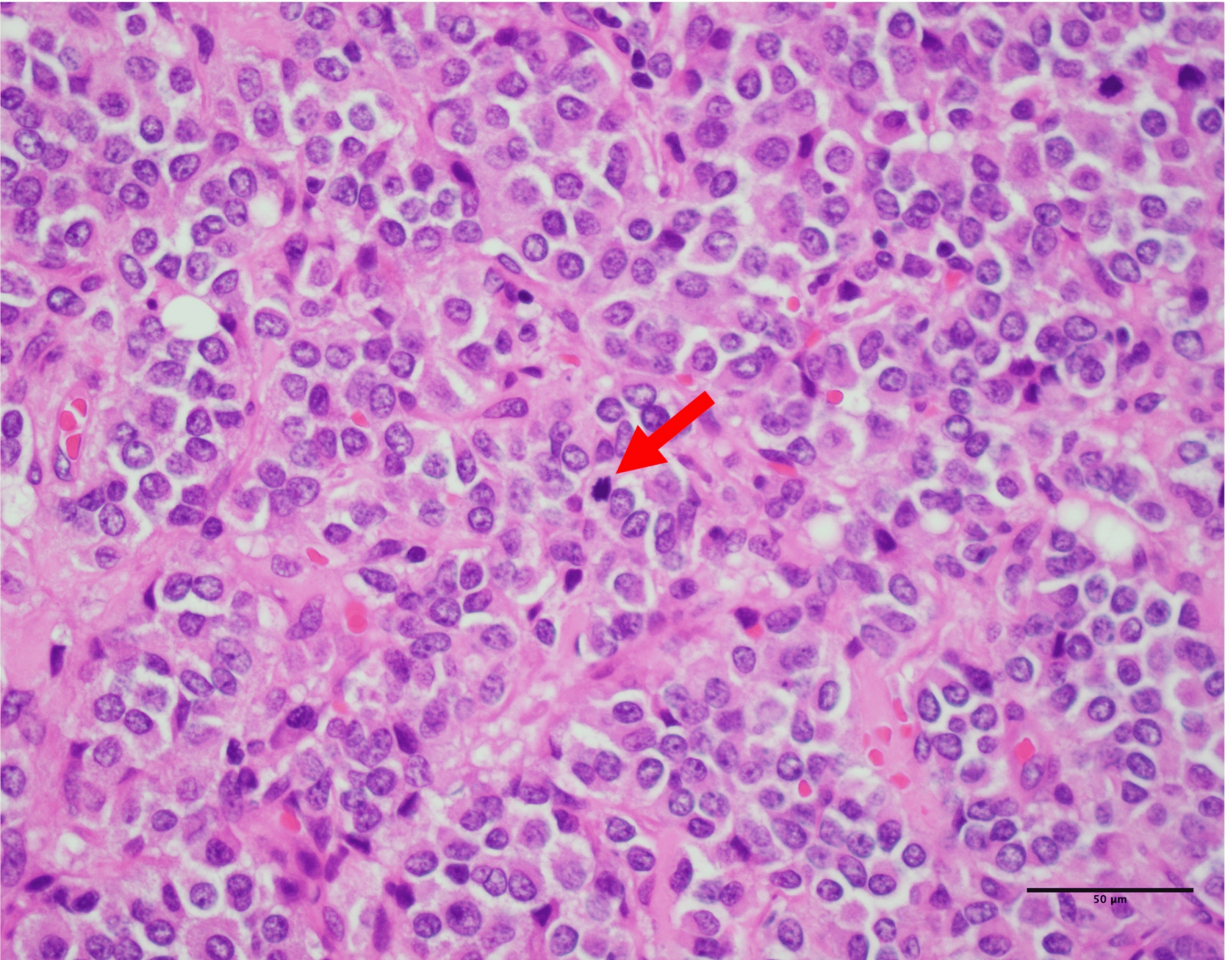
Histopathologic findings. Histologic section showing proliferating polygonal cells with a minor population of cells with short spindle-shaped nuclei, along with scattered rosettes, granular nuclear chromatin, and mitotic figures (arrow) (×40).

On histologic examination of the permanent sections, the tumor showed a proliferation of small- to medium-sized polygonal cells, admixed with a minor component of cells with short spindle-shaped nuclei. Scattered rosette-like structures were present. The nuclei were round to nearly round, with granular chromatin and small or inconspicuous nucleoli. Four mitotic figures were identified per 10 high-power fields. No large geographic areas of necrosis were observed, and lymphocytic infiltration was scant (Figure 5).

**Figure 6:**
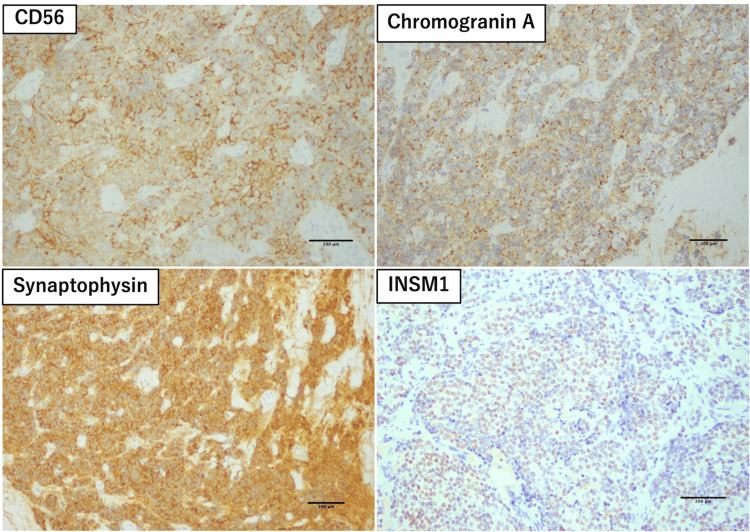
Immunohistochemical findings. Immunohistochemical staining showing diffuse positivity for neuroendocrine markers, including CD56 (neural cell adhesion molecule), chromogranin A, synaptophysin, and insulinoma-associated protein 1 (INSM1) (×10).

Immunohistochemical staining showed diffuse positivity for neuroendocrine markers, including CD56, chromogranin A, synaptophysin, and insulinoma-associated protein 1 (INSM1) (Figure 6) with a Ki-67 labeling index of approximately 20%. CD5, CD117 (c-KIT), p63 (TP63), CK5/6, and CD20 were all negative, and no infiltration by immature T lymphocytes positive for CD99 or TdT was identified.

These findings supported a final diagnosis of thymic AC. The patient received concurrent chemoradiotherapy consisting of weekly carboplatin (CBDCA) plus paclitaxel (PTX) and external beam radiotherapy (2 Gy per fraction, total dose 54 Gy). The patient has remained clinically stable for four years to date.

## Discussion

Thymic AC is defined by increased mitotic activity (2-10 mitoses per 2 mm², equivalent to approximately 10 high-power fields in most microscopes) compared with TC and/or the presence of small foci of necrosis [10]. The Ki-67 labeling index, reflecting the expression of the nuclear protein Ki-67 in proliferating cells, is usually higher than that in TC and may reach approximately 30%. However, it is considered an adjunctive parameter and is not used as the primary criterion to distinguish TC from AC [2,6]. In our case, a mitotic count of 4 per 10 high-power fields, a Ki-67 index of approximately 20%, and the absence of large confluent areas of necrosis were consistent with AC.

Type A thymoma is composed of spindle- or oval-shaped epithelial cells with relatively uniform nuclei and few or no accompanying immature T lymphocytes [2,5]. In limited samples such as small biopsies or frozen sections, thymic AC may resemble type A thymoma in that the tumor cells are relatively monomorphic, may include a spindle cell component, and lack a background of immature lymphocytes [5]. In our case, the frozen sections showed a proliferation of small- to medium-sized tumor cells, partly spindle-shaped, without a lymphocyte-rich background; together with the patient’s history of atypical type A thymoma, this led to an intraoperative diagnosis of recurrence, while the features suggestive of neuroendocrine differentiation were not fully appreciated.

Imprint cytology is a simple, rapid, and inexpensive technique that is commonly used as an adjunctive method for intraoperative diagnosis [11]. Compared with frozen sections, imprint cytology has the advantage of better preservation of nuclear and cellular details, allowing clearer assessment of critical nuclear features while minimizing freezing artifacts [9,12]. In the present case, imprint cytology clearly demonstrated neuroendocrine features, including salt-and-pepper chromatin and rosette formation. Had these cytologic features been fully taken into account during intraoperative consultation, a more cautious intraoperative diagnosis, such as TET, suspicious for neuroendocrine differentiation, could have been rendered.

Immunohistochemistry plays a pivotal role in confirming neuroendocrine differentiation and in excluding other TETs such as thymomas and thymic carcinomas [5]. tNENs typically show strong and diffuse expression of neuroendocrine markers, including chromogranin A, synaptophysin, CD56, and INSM1. CD56, also known as neural cell adhesion molecule, is one of the most commonly used markers of neuroendocrine differentiation [13]. INSM1 has emerged as a relatively novel and more sensitive nuclear immunohistochemical marker, with particular utility in the diagnosis of high-grade neuroendocrine neoplasms [14]. By contrast, type A thymoma usually shows diffuse positivity for broad-spectrum cytokeratins (e.g., pan-cytokeratin, AE1/AE3), whereas neuroendocrine markers are typically negative. In addition, thymomas characteristically contain at least a small population of immature T lymphocytes expressing TdT, CD3, and CD99, even though these cells may be relatively sparse [2,5]. Thymic carcinoma, particularly thymic squamous cell carcinoma, is characterized by the expression of CD5, CD117, p63, and CK5/6-markers that were all negative in the present case [2,5].

From this case, several important points can be highlighted. When encountering an anterior mediastinal mass, the possibility of a tNEN should always be considered, especially when histology shows organoid, trabecular, or rosette-like architectures with finely granular “salt-and-pepper” chromatin [6]. Performing and evaluating imprint cytology in parallel with frozen sections should be routinely considered in the rapid assessment of TETs, because this technique can provide valuable information on nuclear and cellular details. Finally, the use of a comprehensive immunohistochemical panel is essential for distinguishing thymic TC/AC from thymoma and other TETs.

## Conclusions

We report a case of thymic AC in a patient with a prior history of atypical type A thymoma. On frozen section, the tumor closely mimicked type A thymoma, whereas imprint cytology clearly revealed the nuclear and architectural features of a neuroendocrine neoplasm. Immunohistochemistry confirmed neuroendocrine differentiation and excluded thymoma and other thymic carcinomas. This case highlights the importance of integrating frozen-section histology, imprint cytology, and immunohistochemistry for accurate diagnosis of TETs, as well as the significant role of imprint cytology in avoiding misclassification of thymic neuroendocrine neoplasms as thymoma.
